# Early-stage atherosclerosis in poloxamer 407-induced hyperlipidemic mice: pathological features and changes in the lipid composition of serum lipoprotein fractions and *sub*fractions

**DOI:** 10.1186/s12944-016-0186-7

**Published:** 2016-01-22

**Authors:** Tatyana A. Korolenko, Thomas P. Johnston, Fedor V. Tuzikov, Natalia A. Tuzikova, Alexandr B. Pupyshev, Victor K. Spiridonov, Natalya V. Goncharova, Igor V. Maiborodin, Natalia A. Zhukova

**Affiliations:** Institute of Physiology and Fundamental Medicine, Siberian Branch of the Russian Academy of Medical Sciences, Timakov St. 4, Novosibirsk, 630117 Russia; Division of Pharmaceutical Sciences, School of Pharmacy, University of Missouri-Kansas City, Rm. 4243, HSB, 2464 Charlotte Street, Kansas City, MO 64108-2718 USA; Boreskov Institute of Catalysis, Siberian Branch of the Russian Academy of Sciences, Novosibirsk, Russia; Novosibirsk State University, Novosibirsk, Russia; Vorozhtzov N.N. Institute of Organic Chemistry, Siberian Branch of the Russian Academy of Sciences, Novosibirsk, Russia; Novosibirsk State Medical University, Novosibirsk, Russia; Siberian Division of the Russian Academy of Sciences, Institute of Chemical Biology and Basic Medicine, Novosibirsk, Russia

**Keywords:** Atherosclerosis, Cysteine protease (cathepsin B), Dyslipidemia, Lipoprotein fractions and *sub*fractions, Liver enzymes, Lysosomes, Macrophages, Poloxamer 407

## Abstract

**Background:**

The aims of this study were to evaluate the effect of poloxamer 407 administration on atherogenic serum lipoprotein fractions and *sub*fractions associated with cholesterol, triglycerides and phospholipids, as well as the onset of early atherosclerosis, in mice.

**Methods:**

Mice were administered either sterile saline or poloxamer 407 (to induce a dose-controlled hyperlipidemia) for 1 month and then sacrificed at 1, 4 and 10 days after the last dose of poloxamer 407. Systolic and diastolic blood pressure, the activity of a cysteine protease (cathepsin B) in cardiac and liver tissue, and histological/morphological examination of heart and liver specimens was performed for each group of mice at each time point. Lastly, small angle X-ray scattering was utilized to analyze the lipoprotein fractions and *sub*fractions associated with cholesterol, triglycerides and phospholipids for both groups of mice at each time point. Statistical analysis was performed using one-way, analysis-of-variance with *post hoc* analysis to determine significantly different mean values, while correlation analysis employed the Spearman test.

**Results:**

Poloxamer 407-treated mice revealed significant hyperlipidemia, moderately elevated blood pressure, general lipidosis in liver cells, increased cysteine protease activity in heart tissue, and contractile-type changes in cardiomyocytes. Similar to humans, the onset of atherosclerosis in poloxamer 407-treated mice was characterized by a steady increase in serum low-density, intermediate-density and very-low-density lipoprotein fractions, as well as very-low-density lipoprotein *sub*fractions.

**Conclusions:**

We would propose that the sustained elevation of serum atherogenic lipoprotein fractions and *sub*fractions induced by the administration of poloxamer 407 to mice resulted in the morphological changes we observed in both heart and liver cells, which are suggested to precede atherosclerosis, since this is a well-established mouse model of atherosclerosis. Since most of the cellular, biochemical and physiological changes documented in the present study using poloxamer 407-treated mice are related to the symptoms of early atherosclerosis in humans, it is suggested that the poloxamer 407-induced mouse model of hyperlipidemia and atherosclerosis might prove beneficial as an experimental animal model with which to evaluate the pathological features observed in early-stage atherosclerosis.

## Background

Early diagnosis and changes associated with atherosclerosis are important to clinical medicine [1]. Different animal models of hyperlipidemia and atherosclerosis (diet, genetic, chemically-induced, etc.) have been developed [2, 3]. However, all animal models of hyperlipidemia and atherosclerosis have advantages and disadvantages. [4]. A particular chemically-induced mouse model of hyperlipidemia and atherosclerosis involves the chronic administration of a block copolymer called poloxamer 407 (P-407) to mice of either sex. This model produces significant dyslipidemia (for approximately 4 days following a single dose) and subsequent atherosclerosis accompanied by damage to heart vessels [5]. The maximum serum total cholesterol and triglyceride (TG) concentrations obtained after a single dose of P-407 are dose-dependent. The P-407-mediated mouse model of hyperlipidemia and atherosclerosis is a well-established mouse model of atherogenesis, with aortic atheroma formation commencing at about 1 monthnth and attaining maximum lesion size and lesion numbers at approximately 4 months following the initiation of P-407 treatment [6]. To maintain an atherogenic serum lipid profile, P-407 must be dosed approximately every 3 days for the 4-month period, although implantable, controlled-release osmotic pumps represent an alternative to repetitive intraperitoneal injections of P-407.

In the present study, we induced early atherosclerotic lesion formation in mice by using repeated P-407 administration at a relatively low dose for one month to produce a sustained atherogenic serum lipid profile. One advantage of the present study was the use of small-angle X-ray scattering (SAXS) for the simultaneous evaluation of serum lipoprotein-cholesterol (LP-C), lipoprotein-triglyceride (LP-TG) and lipoprotein-phospholipids (LP-PL), which allowed for the determination of the fractional and *sub*fractional composition of LP-C, LP-TG and LP-PL during the onset of experimental atherosclerosis. The primary motivation for lipoprotein fractionation and subsequent analysis of the fractions into *sub*fractions has been an attempt to better treat patients at risk for coronary artery disease resulting from atherosclerosis [7]. In brief, advanced lipoprotein tests (those tests that separate the lipoprotein fractions into *sub*fractions) have been used in four basic ways: 1) to enhance the accuracy of atherosclerosis risk prediction, 2) to enhance the accuracy of outcome prediction, 3) to assist in treatment selection and dose adjustment, and 4) to counsel first-degree relatives of patients with atherosclerosis [7]. A variety of analytical methods have been utilized to achieve *sub*fractionation of lipoproteins, which include, but are not limited to, density gradient ultracentrifugation, gradient gel electrophoresis, immunoaffinity chromatography, ion mobility and 2-dimensional gel electrophoresis [7]. However, standardization between all of these techniques and the numerous fractions and *sub*fractions they produce is critically needed before their widespread clinical application becomes routine practice in cardiovascular medicine [7].

Therefore, the first aim of this study was to evaluate the effect of prolonged (1 mo) P-407 treatment on alterations in the serum concentrations of lipoprotein fractions and *sub*fractions associated with cholesterol, triglyceride and phospholipid in mice, as well as to more precisely describe the time-course over which the elevated serum fractions and *sub*fractions of LP-C, LP-TG and LP-PL returned to baseline levels following discontinuation of P-407 treatment. Our second aim was to determine any cellular, biochemical, and/or pathophysiological changes in this mouse model that are commonly associated with the atherogenic cascade, e.g., changes in blood pressure, cell structure/morphology, lipid movement and storage/sequestration, blood glucose, the activity of serum enzymes predictive of liver function (i.e., ALT and AST) and the activity of a cysteine protease (cathepsin B) in cardiac and hepatic tissue. Our overarching goal was to more fully characterize the pathological changes associated with early-stage atherosclerosis in the P-407-induced mouse model of hyperlipidemia and atherosclerosis.

This article reports the progressive increase in serum low-density, intermediate-density and very-low-density lipoprotein fractions, as well as very-low-density lipoprotein *sub*fractions in P-407-treated mice, which is commonly observed in human patients with developing coronary artery atherosclerotic heart disease. In addition, we report in this article that P-407-treated mice, in addition to manifesting hyperlipidemia, exhibited moderately elevated blood pressure, general lipidosis in liver cells, increased cathepsin B activity in cardiac tissue, and contractile-type morphological changes in cardiomyocytes; all changes frequently associated with the development of early atherosclerosis in humans.

## Results

### Effect of repeated P-407 administration on blood pressure in mice

**Fig. 1:**
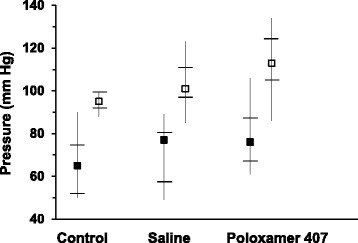
Systolic (□) and diastolic (■) blood pressure in mice following prolonged (1 mo) administration of poloxamer 407. Blood pressure measurements were obtained 24 h following discontinuation of P-407 treatment and 8 h prior to sacrifice. Filled and open square symbols represent the median values, whereas the upper and lower point of the vertical lines (“whiskers”) represent the maximum and minimum blood pressure reading, respectively. Horizontal hash marks (lines) through the vertical lines represent the values of the 1st-quartile, or 25th percentile (*lower*) and 3rd-quartile, or 75th percentile (*upper*)

Following 1 month of P-407 treatment, there was a slight upward trend in both the systolic and diastolic blood pressure in mice when compared to controls treated with saline (Fig. 1). Mean values for both the systolic and diastolic blood pressure followed the rank order; P-407-treated mice > saline-treated controls > non-treated controls and demonstrated that even the treatment protocol (2 injections per week) results in an upward trend in blood pressure as demonstrated by an elevated systolic and diastolic blood pressure in saline-treated controls versus non-treated controls (Fig. 1).

### Effect of repeated P-407 administration on serum protein, glucose, ALT and AST

Both serum total protein (*p* < 0.01) and ALT activity (*p* < 0.05) were significantly elevated, relative to controls, 24 h following cessation of P-407 treatment for 1 month (Table 1). The serum glucose concentration was significantly (*p* < 0.05) reduced at 4 days after the last dose of P-407 when compared to controls (Table 1). There was a positive correlation between total serum protein and total cholesterol (Spearman correlation coefficient = 0.722, t = 2.95, *p* < 0.02) and total serum protein and TG (Spearman correlation coefficient = 0.687, t = 2.67, *p* < 0.03). Additionally, there was a positive correlation between serum non-HDL cholesterol and ALT activity (Spearman correlation coefficient = 0.783, t = 3.55, *p* < 0.01), non-HDL-cholesterol and AST activity (Spearman correlation coefficient = 0.764, t = 3.35, *p* < 0.01), and total serum cholesterol and glucose (Spearman correlation coefficient = 0.692. t = 2.71, *p* < 0.05).

**Table 1:** Time-dependent return of select biochemical parameters to normal concentrations following 1 month of poloxamer 407 administration to mice

Analyte	Control	P-407^1^ (24 h)	P-407^1^ (4 days)	P-407^1^ (10 days)
Total protein (g/l)	64.8 ± 11.1	**120 ± 4.7	61.2 ± 7.4	63.8 ± 0.9
Glucose (mg/dl)	79.5 ± 5.8	82.9 ± 5.0	*54.0 ± 2.2	66.7 ± 3.8
ALT (U/l)	37.8 ± 4.5	*50.1 ± 4.7	28.5 ± 1.9	29.1 ± 1.1
AST (U/l)	144 ± 11.3	146 ± 14.3	152 ± 9.0	165 ± 17.4

^1^Times indicate the time period following the discontinuation of P-407 administration for 1 month

*p < 0.05

**p < 0.01 vs. control

### Serum LP-C, LP-TG and LP-PL concentrations; their fractions and subfractions

**Fig. 2:**
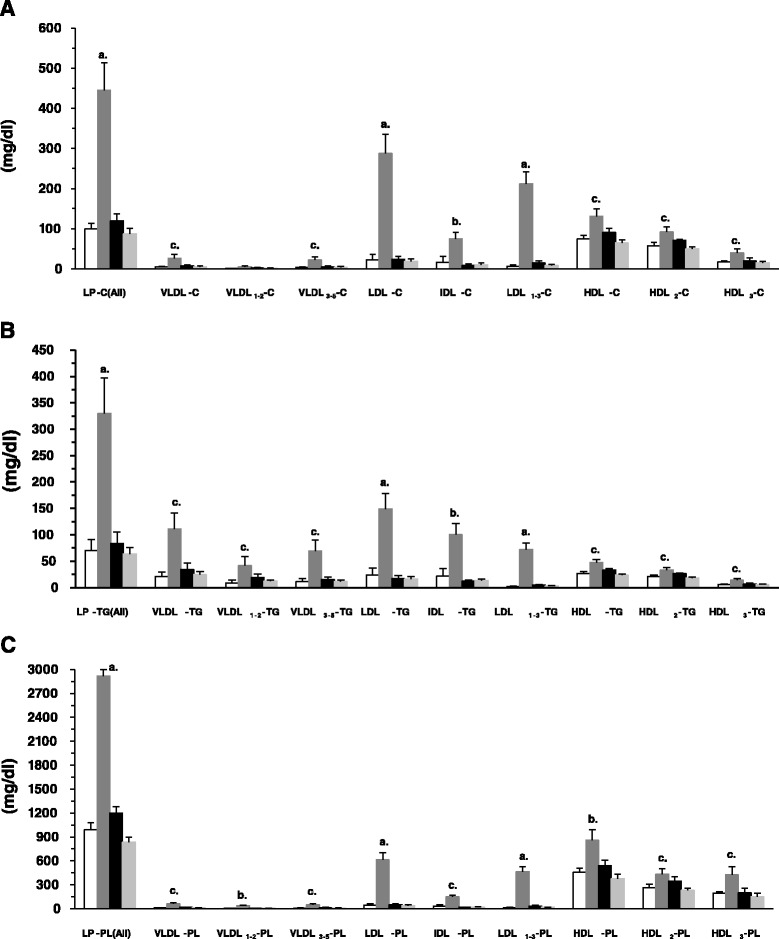
Serum lipoprotein fractions and *sub*fractions associated with cholesterol (LP-C) (**a**) triglycerides (LP-TG) (**b**), and phospholipids (LP-PL) (**c**) in mice following prolonged (1 mo) administration of poloxamer 407. Dark-grey, black and light-grey bars (bars 2, 3 and 4 in each group of 4 bars) indicate the concentration of either LP-C, LP-TG or LP-PL at 1, 4 and 10 days after stopping P-407 administration, respectively, whereas the open or unfilled bar (bar 1 in each group of 4 bars) indicates the concentration of either LP-C, LP-TG or LP-PL in saline-treated (control) mice. **a**, **b** and **c** above bars indicate *p* < 0.001, *p* < 0.01, and *p* < 0.05, respectively, vs. control

Figures 2a, b and c show all of the lipoprotein fractions and *sub*fractions associated with cholesterol (Fig. 2a), TG (Fig. 2b) and phospholipids (Fig. 2c) determined at 24 h, 4 d and 10 d following the last dose of P-407, which was administered to mice for 1 month. Some overall general trends are noteworthy. To begin, if one considers the total serum LP-C, LP-TG and LP-PL (i.e., the first group of 4 bars in Figs. 2a, b and c, respectively), each lipid was significantly (*p* < 0.001) elevated over control values at 24 h. By 4 days post-treatment, all three lipids had decreased to serum concentrations that were approximately equal to control values. As explained in the Methods section, the K_ch_ for P-407-treated mice (24 after stopping P-407 administration) was 2.3 compared to 0.37 for control mice, which is a greater than 6-fold increase in the value of K_ch_ for P-407-treated mice and provides, based on serum concentrations of lipoproteins alone, additional validation as to the degree of atherogenicity and propensity for atherosclerosis in the P-407 model.

As it pertains to only non-HDL lipoprotein fractions and *sub*fractions associated with *cholesterol* (Fig. 2a), VLDL, LDL and IDL remain significantly elevated 24 h after P-407 treatment is stopped when compared to controls, while HDL lipoprotein fractions and *sub*fractions appeared to return to normal levels in a more gradual fashion.

In contrast, lipoprotein fractions and *sub*fractions associated with *triglyceride* (Fig. 2b), show that, in general, the VLDL fraction and *sub*fractions contained more TG. This would seem to support our previous findings, which have demonstrated that the elevation in serum TG is more pronounced than the rise in serum total cholesterol in P-407-treated mice and that most of the TG is associated with the VLDL fraction [6]. Additionally, the LDL, IDL and HDL lipoprotein fractions and *sub*fractions associated with TG were significantly greater than corresponding mean values for controls 24 h after the last dose of P-407 (Fig. 2b).

Finally, there was a significant elevation in the HDL lipoprotein fraction (*p* < 0.01), and *sub*fractions (*p* < 0.05) associated with *phospholipid* (Fig. 2c) 24 h after the last dose of P-407 relative to controls. When compared to corresponding mean values for controls, it should also be noted that there was a significant increase in the LDL lipoprotein fraction (*p* < 0.001) and *sub*fraction (LDL_1–3_) (*p* < 0.001), as well as the IDL lipoprotein fraction (*p* < 0.05), associated with *phospholipid* 24 h after stopping P-407 administration (Fig. 2c).

### Degree of lysosomal membrane permeability

**Fig. 3:**
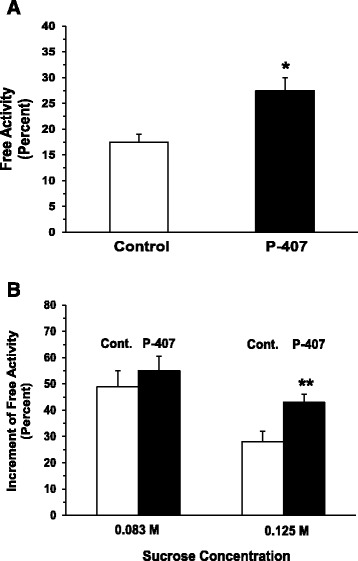
The effect of prolonged (1 mo) administration of P-407 in mice on liver lysosomal membrane permeability as suggested by (**a**) the release of β-galactosidase, which is expressed as the free activity of β-galactosidase as a percent of the total activity, and (**b**) hypoosmotically-induced membrane rupture, which is expressed as the increment of free activity of β-galactosidase. * and ** indicates *p* < 0.05 and *p* < 0.01, respectively, vs. control

Liver lysosomes examined 24 h after the last dose of P-407 revealed a significant (*p* < 0.05) increase in the free activity of β-galactosidase (Fig. 3a), as well as a significant (*p* < 0.01) increase in their susceptibility to hypoosmotic treatment in vitro when 0.125 M sucrose was used (Fig. 3b).

### Cathepsin B activity in liver and heart tissue

**Fig. 4:**
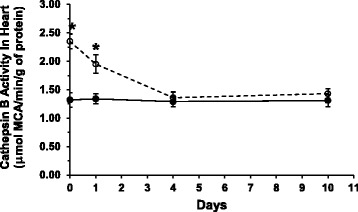
Cathepsin B activity in heart tissue after prolonged poloxamer 407 administration in mice. Data points at 1, 4 and 10 days for both P-407-treated (dashed line with open circles) and control (solid line with filled circles) mice represent the elapsed time period after stopping P-407 dosing for 1 month. *indicates *p* < 0.01 vs. control

The specific activity of cathepsin B in liver tissue increased modestly 24 h after stopping P-407 treatment for 1 month, although it did not reach statistical significance when compared to control (data not shown). In contrast, the specific activity of cathepsin B in heart tissue remained significantly (*p* < 0.01) elevated relative to control for as long as 24 h after the last dose of P-407 (Fig. 4); however, the enzyme activity began to normalize and returned to normal (control) values by day 4 following discontinuation of P-407 treatment (Fig. 4).

### Morphological evaluation of liver and heart cells

#### Light microscopy of liver cells

**Fig. 5:**
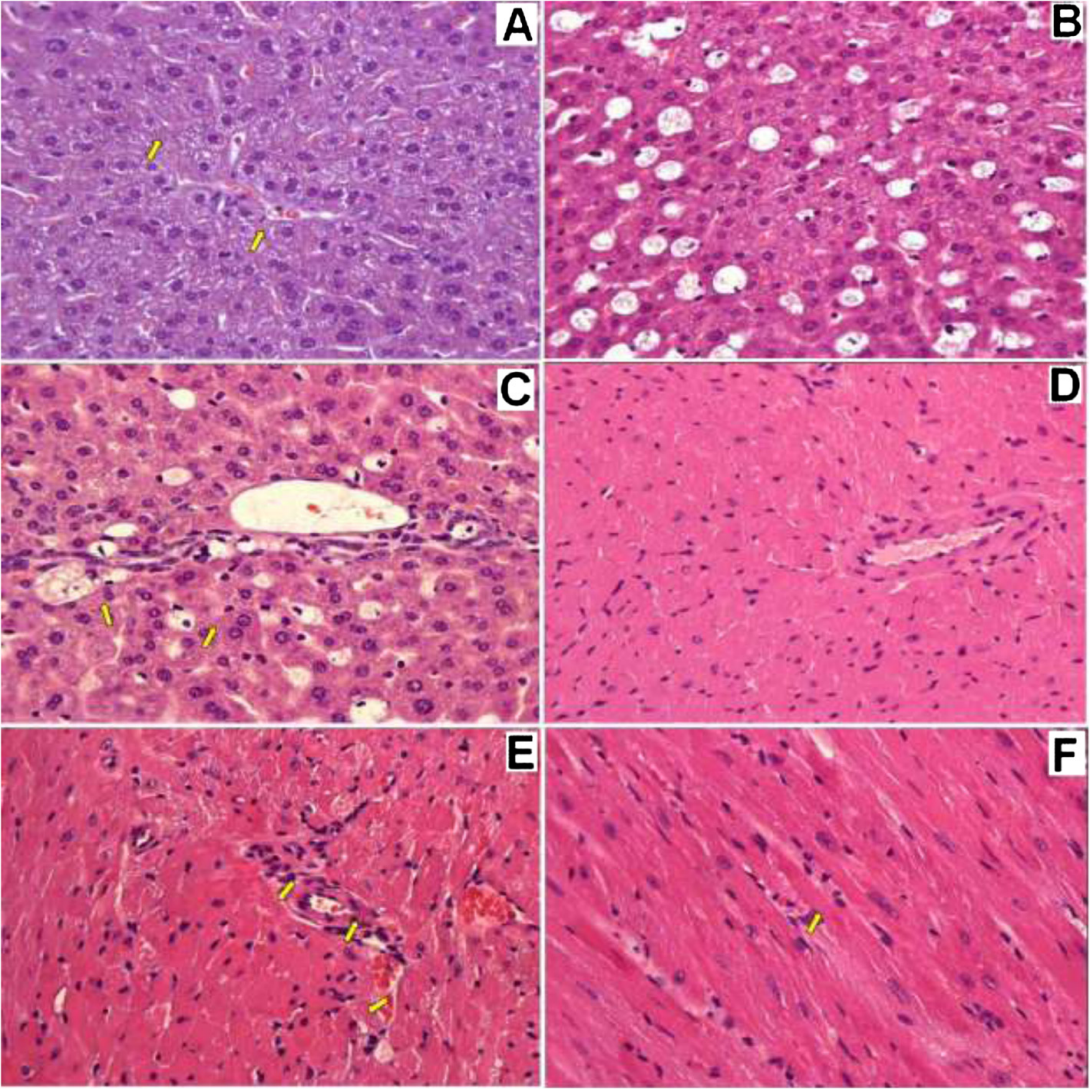
Light microscopic findings of liver (**a**, **b**, **c**) and heart (**d**, **e**, **f**) of mice following prolonged (1 mo) administration of poloxamer 407. All specimens were stained with hematoxylin and eosin at the magnification indicated. **а** Liver of control mice, 400 X. **b** Liver of mice 4 days after discontinuation of P-407 administration. Hepatocytes and macrophages exhibited ‘foamy’ cytoplasm, 400 X. **с** Liver of mice 10 days after discontinuation of P-407 administration. Increased number of ‘foamy’ macrophages are visible, which appear to be localized in sinusoids and in the periportal zone, 400 X. **d** Myocardium of control mice, 200 X. **e** Myocardium of mice 24 h after discontinuation of P-407 administration. Swollen vessel walls of the muscle-elastic type are visible, together with localized changes of contracture-type cardiomyocytes shown with arrows, 400 X. **f** Myocardium of mice 24 h after discontinuation P-407 administration. Perivascular edema in the walls of blood vessel in interstitial tissue with groups of cells overloaded with lipids (*arrow*), 400 X

**Fig. 6:**
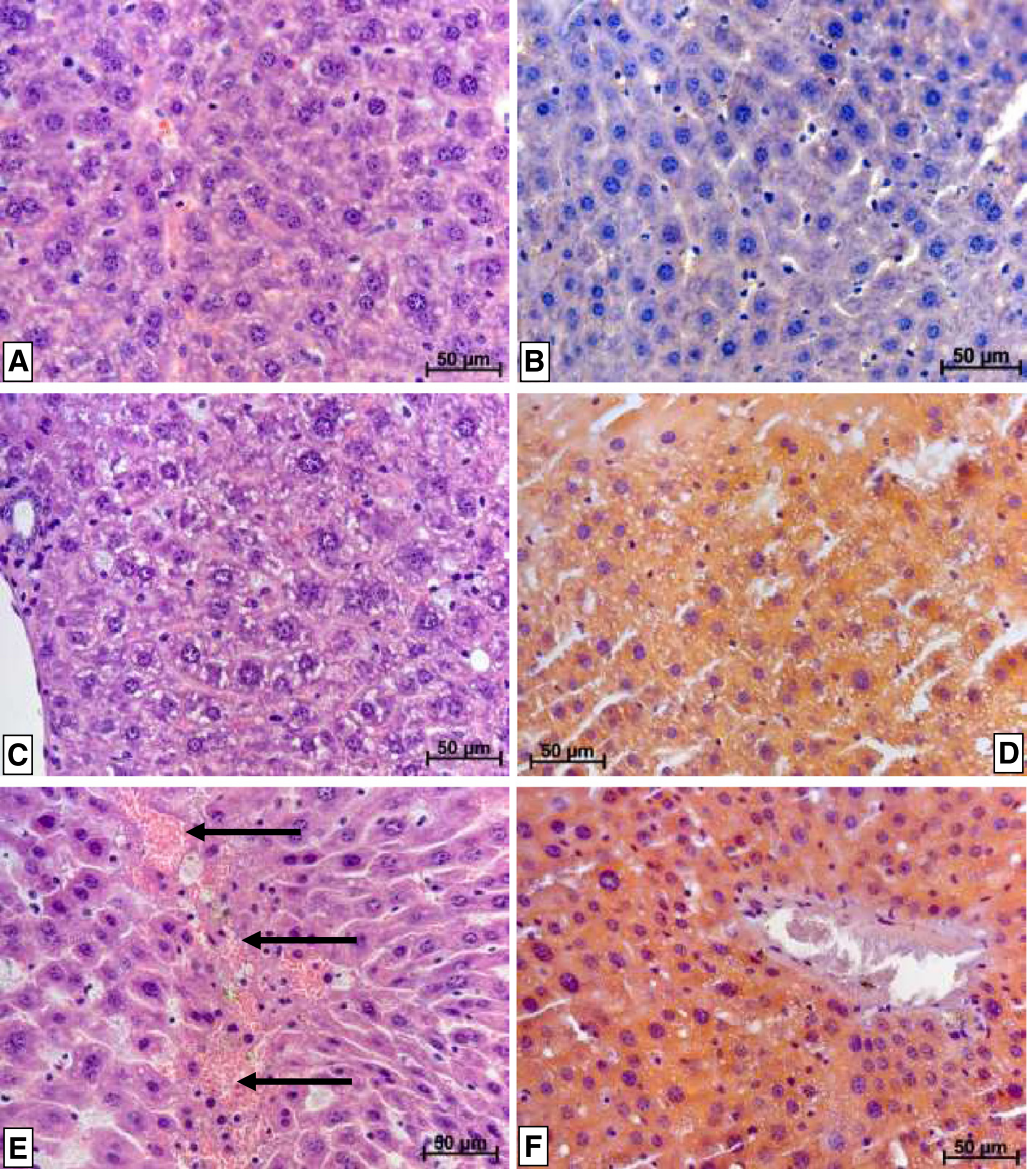
Lipid staining in liver of mice after 1 month of P-407 administration. H&E = hematoxylin and eosin staining, while S&H = Sudan III and hematoxylin staining. Magnification is represented by linear distance according to the scale on each histology slide (**a**) through (**f**). **a** Liver of control mice, H&E. **b** Liver of control mice, S&H. **c** Liver of mice 24 h after discontinuation of P-407 administration for 1 month. Mild vacuolar dystrophy of hepatocytes, together with necrosis of some hepatocytes, H&E. **d** Liver of mice 24 h after discontinuation of P-407 administration for 1 month. All hepatocytes are filled with small and medium-sized lipid droplets, thus displaying significant lipid dystrophy, S&H. **e** Liver of mice at 4 days after discontinuation of P-407 administration for 1 month. Hepatocytes are significantly enlarged and homogenously stained. There is diffuse leukocyte infiltration visible. The portion of hepatocytes demonstrating necrosis appear to have incorporated erythrocytes (arrows), H&E. **f** Liver of mice 4 days after discontinuation of P-407 administration for 1 month. All hepatocytes are filled with small lipid droplets, thus displaying significant lipid dystrophy, S&H

Liver cells obtained from mice 24 h after discontinuation of P-407 treatment revealed discomplexation of liver cells, signs of local intrahepatic cholestasis, (Fig. 5b and c) and venous stasis (Fig. 5c) compared to controls (Fig. 5a). An increase in the size of the sinusoids was noted 4 days after discontinuation of P-407 treatment (Fig. 5b). Hepatocytes were increased in size and exhibited a cytoplasm that appeared “foamy” (Fig. 5b and c). Numerous macrophages, which also appeared to contain a “foamy” substance, were observed in enlarged sinusoids and in periportal zones. This was particularly evident by day 4 following discontinuation of P-407 treatment (Fig. 5b). Similar changes were also noted 10 days after stopping P-407 treatment (Fig. 5c). Additionally, in some cells, vesicular lipid dystrophy was noted. This is particularly evident with Sudan III and hematoxylin stain at both 24 h and 4 days following the discontinuation of P-407 treatment (Fig. 6d and f) when compared to control (Fig. 6b). Finally, hepatocytes appeared to have been mildly infiltrated with lymphocytes, which seemed to lessen 10 days following cessation of P-407 treatment. In summary, liver injury in P-407-treated mice was characterized by lipid storage in liver cells (especially in macrophages), discomplexation of liver cells and increased cholestasis (Figs. 5b, c and 6c–f).

#### Light microscopy of cardiomyocytes

When compared to cardiomyocytes isolated from controls (Fig. 5d), changes observed in P-407-treated mice consisted primarily of damage to contractile-type cardiomyocytes (Fig. 5e), which is typical for lipidosis (Figs. 5e and f). There was swelling of the muscle layer inside of membrane vessels, together with adherence of fibrin and erythrocytes to the luminal wall of vessels (Fig. 5e). Xanthoma cells had infiltrated the perivascular zones. Twenty-four hours after the last dose of P-407, xanthoma cells had also infiltrated the matrix area, and, by day 4, these same type of cells were observed in the endocardial space.

## Discussion

Diagnosis of early atherosclerosis is important for prophylactic treatment strategies to combat this disease [8, 9]. The use of animal models to provide new approaches for improving the diagnosis and treatment of cardiovascular diseases is extremely important to cardiovascular medicine [10, 11]. The advantage of the dose-dependent, pharmacologically-induced mouse model of hyperlipidemia and atherosclerosis used in the present work is that it reliably recapitulates the phenotypic signatures associated with the pathogenesis of human atherosclerosis (hyperlipidemia, atheroma formation, cardiac muscle and vessel injury, etc.) [6]. Moreover, high levels of circulating LP-cholesterol in the P-407 model partially reproduces familial hypercholesterolemia and familial combined hyperlipidemia (inherited disorders associated with increased circulating LDL-cholesterol and severe atherosclerosis) in humans with poor prognosis and treatment [12]. Thus, this model has proven beneficial for the study of various stages involved with hyperlipidemia-induced atherosclerotic lesion formation and has been utilized to effectively screen the potency and efficacy of several classes of anti-hyperlipidemic drugs [13, 14], as well as demonstrate the effectiveness of atorvastatin to cause regression of established atherosclerotic lesions [13].

The present work demonstrated that the atherogenic serum lipid profile induced with P-407 treatment in mice is reversible once P-407 administration is discontinued. One of the main findings in our study was that repetitive dosing of P-407 in mice induced a hyperlipidemic state that more closely resembles hyperlipidemia observed in the early stages of atherosclerosis in humans, with simultaneous development of liver lipidosis and early signs of heart injury. Importantly, because it is known that the total serum cholesterol concentration alone does not accurately predict the risk of cardiovascular disease in many patients [15], we simultaneously evaluated all of the fractions and *sub*fractions of LP-C, LP-TG and LP-PL by employing SAXS analysis [16, 17].

Using SAXS, one of our goals in the present investigation was to quantify the serum concentrations of lipoprotein fractions and *sub*fractions associated with cholesterol, triglyceride and phospholipid in P-407 treated mice relative to controls. These findings will now be discussed in relation to the P-407-induced mouse model of atherogenesis. In each and every case (with the exception of the VLDL_1–2_*sub*fraction associated with cholesterol), the atherogenic (i.e., VLDL_1–2_, VLDL_3–5_, IDL and LDL_1–3_) and antiatherogenic (i.e., HDL_2_ and HDL_3_) lipoprotein fractions and *sub*fractions associated with cholesterol, TG and PL in mice were significantly greater 24 h after discontinuing P-407 administration when compared to controls. Irrespective of the serum concentrations of all lipoprotein fractions and *sub*fractions in P-407-treated mice relative to control mice, it is extremely important to emphasize that despite the presence of antiatherogenic HDL_3_, and especially HDL_2_, the capacity for the atherogenic lipoprotein fractions and *sub*fractions to induce the formation of aortic atherosclerotic lesions in P-407-treated mice is not completely inhibited by HLD_2_ and HDL_3_, since this is a well-established mouse model of hyperlipidemia and atherogenesis [6, 13, 18, 19]. Additionally, as it relates to the atherogenicity of lipoprotein fractions and *sub*fractions, the intensity of the atherogenic response in P-407-treated mice is greatly enhanced by the enormous increase in the LDL fraction and *sub*fractions, which undergo spontaneous lipid peroxidation to form oxidized-LDL (ox-LDL) in vivo [20]; a highly-atherogenic lipoprotein particle capable of easily entering the arterial intima through transendothelial migration [21].

It was interesting to note in our work that serum non-HDL-cholesterol exhibited a strong association with both serum ALT and AST activity (Spearman correlation coefficients were approximately equal to 0.8), because Jiang et al. has recently shown a U-shaped relationship between serum non-HDL-cholesterol and elevated transaminases (both ALT and AST) in humans [22]. Jiang et al. suggest that there may be a strong association between lipoprotein metabolism and liver diseases/injury when serum non-HDL-cholesterol is either extremely low, or extremely high. These authors determined that the same U-shaped relationship also existed for a more narrow portion or component of the total non-HDL-cholesterol; namely, between just serum LDL-C alone and serum transaminases [22]. While an association does not prove causality, nevertheless, the extremely high non-HDL-cholesterol fractions and *sub*fractions seen with P-407-treated mice may be associated with the elevations in serum ALT and AST we observed in the present study.

Jiang et al. suggest that disorders in lipoprotein metabolism may potentially lead to hepatic injury, whereas chronic liver disease may also interfere or impair lipoprotein production. Interestingly, we have previously demonstrated that the activity of key enzymes and, more specifically, lipases involved with lipid metabolism (e.g., hepatic lipase, lipoprotein lipase, endothelial lipase and pancreatic lipase) are either partially, or fully, inactivated in vivo in P-407-treated mice [23, 24]. This would clearly represent a disorder, or disturbance, in lipid and lipoprotein metabolism, which, according to Jiang et al., could potentially cause hepatic injury and hence, contribute to an elevation in ALT and AST activity in the serum of P-407-treated mice. When taken together with the morphological changes in specific cells of liver tissue (i.e., liver injury resulting from elevated lipid influx and sequestration, since P-407 itself is not hepatotoxic), which we have documented in P-407-treated mice, it may be that hepatic lipoprotein production is potentially altered/perturbed due to these pathophysiological changes as suggested by Jiang et al. This finding would lend support to their hypothesis of a bidirectional relationship between lipoprotein metabolism and liver injury/disease [22].

Early, but subtle, changes in cardiomyocytes resulting from prolonged administration of P-407 in mice were documented with light microscopy in the present study. Changes to contractile-type cardiomyocytes in coronary vessels were observed, which usually precedes atheroma formation. In fact, we have previously reported these same changes to contractile-type cardiomyocytes and similar, but more pronounced, coronary vessel injury following extended (4 mo) P-407 treatment in mice [5].

The potential link between the inflammatory effects of lipemia and the induction of macrophage foam cell formation by triacylglycerol-rich lipoproteins has been shown by others [25, 26]. When inflow and esterification of cholesterol increase and/or its outflow decrease, macrophages are ultimately transformed into lipid-laden foam cells; the prototypical cells formed in atherosclerotic plaque [27]. In the present investigation using light microscopy, we observed numerous liver macrophages containing lipid material (xanthoma cells), which were significantly larger in size than adjacent cells. We would suggest that the increased size of lipid-laden macrophages is potentially due to both intracellular uptake of lipids together with intralysosomal accumulation of P-407, which is similar to what we have previously reported with Triton WR-1339-filled lysosomes of liver cells [28]. Lastly, our microscopic analysis revealed that a majority of hepatocytes were stained with Sudan III, which suggests that protein-bound lipids were present. Since numerous necrotic hepatocytes and intrahepatic cholestasis were also noted in P-407-treated mice, the increase in serum ALT and AST activity we detected would certainly strengthen or support our qualitative microscopic findings with liver cells.

As mentioned above, an increased level of circulating serum lipoproteins and their uptake by liver was followed by lipidosis as a result of intracellular lipid storage. The natural question that arises from such a finding is the identity of the cell compartment(s) in which the lipids accumulate, i.e., inside of lysosomes or outside of lysosomes [29]. In the present work, we have acquired data which would support the involvement of lysosomes in this process, although based on our results with lipid staining using Sudan III, we cannot exclude the possibility of extralysosomal lipid storage. Our measurements of the free activity of lysosomal β-galactosidase suggests an inability of the substrate (4-methylumbelliferyl β-D-galactopyranoside) to reach the lysosomal enzyme until the organelles are deliberately ruptured. Both an increase in the free activity of β-galactosidase and osmotically-induced lysosomal membrane rupture reflect labilization of lysosomal membranes in P-407-treated mice, which we would suggest is related to overloading of lysosomes with lipids [30]. It should be pointed out that the changes in lysosomal membranes in the liver cells of P-407-treated mice may potentially reflect an increase in autophagy (autophagocytosis) of liver cells [31] however, further testing is required to confirm this premise.

Following one month of P-407 administration to mice, we demonstrated an increase in cathepsin B activity in heart tissue 24 h after the last dose of P-407, which seemed to be positively correlated with the morphological changes we observed in contractile-type cardiomyocytes using light microscopy. As suggested by Chen et al. [32], lysosomal membrane permeabilization and subsequent release of lysosomal cathepsin B may contribute to the development of coronary arteritis by activating the endothelial Nlrp3 inflammasome. Moreover, cathepsins regulate chemokine activity and play a role in leukocyte recruitment during protective or pathological inflammation [33].

The rise in heart cathepsin B activity in P-407-treated mice appears to signal the onset of cardiac tissue injury, because atheroma formation normally commences at approximately 1 month after initiating P-407 treatment in this mouse model of atherogenesis [6]. In fact, it has previously been reported that both the number and size of atherosclerotic lesions formed in this animal model typically attains a maximum at 4 months after beginning P-407 administration [6]. It should also be noted that we have reported a much greater increase in the activity of cathepsin B in heart tissue when mice are treated with P-407 for 4 months [5] when compared to the increase in cathepsin B activity following 1 month of P-407 treatment as used in the present study. Thus, this would indicate that the magnitude of the elevation in heart cathepsin B activity is positively correlated with the extent, or degree, of heart injury as a function of time; i.e., the net increase in heart cathepsin B activity and, therefore, resulting cardiac tissue injury, is dependent on the length of time that mice are treated with P-407 and subjected to the resulting atherogenic, inflammatory serum lipid profile, since P-407, by itself, is non-toxic to most cells (including cardiomyocytes) at concentrations utilized in the present study.

## Conclusion

In this investigation, we used an experimental animal model in which relatively small doses of P-407 were repeatedly administered to mice for 1 month to achieve a sustained, atherogenic serum lipid profile; specifically, an increase in serum total TG, as well as atherogenic LDL-cholesterol. The P-407-mediated increase in the atherogenic LP-C fraction (LDL_1–3_-C *sub*fraction) and LP-TG (LDL_1–3_-TG *sub*fraction) in serum was followed by a return to normal values by day 4 following discontinuation of P-407 treatment for 1 month. Significant liver lipidosis was shown during all periods studied, with lipid sequestration occurring primarily in liver cells (macrophages and hepatocytes). It is suggested that lipids (and possibly P-407) accumulate inside of lysosomes based on an increase in lysosomal membrane permeability, although both extralysosomal lipid storage and increased autophagy cannot be unequivocally excluded. Heart injury was characterized by morphological changes in cardiomyocytes, as well as an increase in cathepsin B activity. In summary, all of the cellular, biochemical and pathophysiological changes (including an increase in blood pressure) noted in the present study using P-407-treated mice are related to the symptoms of early atherosclerosis. Therefore, the P-407-induced mouse model of hyperlipidemia and atherosclerosis might prove beneficial as an experimental animal model with which to evaluate the pathological changes that occur in humans during the early stages of atherosclerosis. Importantly, this model may potentially allow for the evaluation of new therapeutics aimed at reversing or halting early-stage atheroma formation in humans. These results are extremely relevant to human health, since early medical intervention improves patient outcomes (quality of life, lifespan, etc.) and reduces the overall cost of health care for cardiovascular disease; the number one cause of mortality in Western countries.

## Methods

Male CBA mice having a body mass of 25–30 g were used and obtained from the breeding station of the Institute of Physiology, Siberian Branch of the Russian Academy of Sciences, Novosibirsk, Russia. Poloxamer 407 (P-407) (Pluronic F-127, Sigma) was administered to mice as an intraperitoneal (i.p.) injection at a dose of 300 mg/kg twice per week for one month [6]. The mice were decapitated at 1, 4 and 10 days after the last dose of P-407. Control mice received an equivalent volume (0.5 mL) of saline. Mice were deprived of food, but had free access to water, 15 h before euthanasia. All procedures for agent administration and blood and tissue collection were in accordance with the 8th edition of the Guide for the Care and Use of Laboratory Animals published in 2011 by the United States National Academy of Sciences, and the treatment protocol (animal protocol #9) was also approved by the Animal Care and Use Committee at the Institute of Physiology and Fundamental Medicine of the Siberian Branch of the Russian Academy of Medical Sciences (RAMS) on May 27, 2014. The in vivo experiments were also conducted in compliance with RAMS Ethical Committee Recommendations pertaining to research involving laboratory animals.

The systolic and diastolic blood pressure was measured in mice with a multi-channel, computerized non-invasive blood pressure system for mice and rats (CODA, Kent Scientific Corporation, Torrington, CT, USA) as described previously [5].

### Serum measurements of lipoprotein fractions and subfractions

Serum was obtained after centrifugation of blood samples at 3000 × g for 20 min at 4 °C (Eppendorf Centrifuge 5415R, Germany) and stored at −70 °C until analysis of lipoprotein fractions and *sub*fractions associated with cholesterol (C), triglycerides (TG) and phospholipids (PL) using small-angle X-ray scattering (SAXS). According to Otvos [16], LP fractions can be divided into the following four main classes: high density LP (HDL), low-density LP (LDL), very-low-density LP (VLDL) and chylomicrons, or seven *sub*fractions: HDL_3_, HDL_2_, LDL, intermediate-density LP (IDL), VLDL_3–5_, VLDL_1–2_ and chylomicrons (chylomicrons were not determined by the method used). Interval borders of fractions and *sub*fractions (according to the scale of sizes, r_o_) were the same as previously described by Otvos [16]. The SAXS method for the determination of the fractional and *sub*fractional composition of LPs has been used and validated previously [17]. This method is inexpensive, rapid and capable of determining the relative content of different LP fractions, both as a size distribution of various LP particles and as absolute units of the total concentration of lipid in LP fractions. The total LP-PL fraction was calculated by subtracting the sum of LP-C + LP-TG from the total LP concentration. Using a small sample of serum (0.1–0.25 ml), it was possible to simultaneously assay the fractions and *sub*fractions associated with LP-C and LP-TG, and then readily compute LP-PL. Lastly, while not lipids, we also analyzed the serum for total protein, glucose and the activity of ALT and AST using a biochemical analyzer (Architect С8000; Abbott, USA).

### Relationship of “SAXS-determined” lipoprotein fractions and subfractions to atherogenicity

As stated above, SAXS was utilized to determine the concentration of six lipoprotein fractions and *sub*fractions associated with cholesterol, triglycerides and phospholipids; namely, VLDL_1–2_, VLDL_3–5_, IDL, LDL_1–3_, HDL_2_ and HDL_3_, however, the seventh lipoprotein fraction (chylomicrons = CM) was not measured in the present study. It is important to point out that our method (SAXS) quantifies 7 lipoprotein fractions and *sub*fractions, but, as an example of the complexity involved with standardizing so many lipoprotein fractions and *sub*fractions identified using a variety of analytical techniques, it has been demonstrated that as many as 2 to 38 *sub*fractions can be separated and quantified for LDL alone [34].

Within the ‘SAXS-determined’ VLDL fraction, there are 5 *sub*fractions, with *sub*fractions 1 and 2 (i.e., VLDL_1_ and VLDL_2_) representing small VLDL. VLDL_1_ and VLDL_2_ are atherogenic due to their small size, apolipoprotein C-III content and enriched cholesteryl ester content. *Sub*fractions 3 and 4 represent intermediate/medium size VLDL particles, which are less atherogenic than smaller VLDL_1_ and VLDL_2_, but still able to enter arterial intima and contribute to eventual atheroma formation. Finally, *sub*fraction 5 represents triglyceride-rich, larger VLDL particles, which, like CM, are too large to potentially enter the arterial wall. Within the LDL fraction, there are 3 *sub*fractions. *Sub*fractions LDL_1_ and LDL_2_ are highly atherogenic due to their small size, increased density, ability to migrate across vascular endothelium, apolipoprotein B-100 content, and, in approximately 10-20 % of serum LDL particles, apolipoprotein C-III content. *Sub*fraction LDL_3_ is a larger and less dense LDL particle than LDL_1_ and LDL_2_, and, therefore, slightly less atherogenic than small, dense LDL *sub*fractions LDL_1_ and LDL_2_.

The HDL fraction actually contains three *sub*fractions when analyzed by the SAXS technique and consists of small dense and intermediate/medium size lipoprotein particles. HDL_1_ and HDL_2_*sub*fractions represent the small, dense HDL *sub*fractions and HDL_3_ represents the intermediate/medium size HDL *sub*fraction. The HDL_1_*sub*fraction is not shown in our results (Fig. 2a, b and c), because human and mouse serum typically contains only a trace amount of HDL_1_ [35]. The HDL_2_*sub*fraction contains apolipoprotein A-II and is therefore antiatherogenic and confers the greatest protection against developing coronary artery disease, while *sub*fraction HDL_3_, being an intermediate/medium-sized HDL particle, is generally less protective towards atherogenesis than *sub*fraction HDL_2_.

Lastly, IDL was historically a member of the LDL fraction, but has been reclassified by itself due to its prominent role in atherosclerosis. Because IDL contains both apolipoprotein C-III and is rich in cholesteryl esters, it represents a highly-atherogenic lipoprotein particle [15].

While the P-407-induced mouse model is already a well-documented mouse model of atherogenesis, nevertheless, we also calculated a value known as the cholesterol atherogenic coefficient (K_ch_) in the present study [36]. Briefly, the K_ch_ is defined as (VLDL-C + LDL-C)/HDL-C and serves as a predictor of myocardial ischemia [36]. The higher the value of K_ch_, the higher the risk of atherogenic changes and the development of atherosclerosis. The mean values of VLDL-C, LDL-C and HDL-C (Fig. 2a) for both control mice and P-407-treated mice (24 h after stopping P-407 administration) were used to calculate a representative K_ch_.

### Membrane stability and osmotic sensitivity of lysosomes

In general, methods described by Wang et al. [37] and Deng et al. [38] were used. Lysosomal stability (as reflected by the degree of lysosomal membrane permeability when measuring the release of the lysosomal enzyme, β-galactosidase) was determined, as well as lysosomal membrane susceptibility to hypoosmotic treatments (0.083 M and 0.125 M sucrose solutions) in vitro. Additionally, lysosomal enzyme latency was assessed by measuring the free activity associated with lysosomal β-galactosidase. The enzymatic activity of β-galactosidase was determined using a fluorogenic method against 4-methylumbelliferyl N-acetyl-β-D-galactosaminidepyranoside (Melford Laboratories, England) as the substrate (1 mM, pH 5) [39]. Briefly, 2 % liver homogenate solutions prepared in isotonic 0.25 M sucrose with 1 mM Na-EDTA, pH 7.4, were diluted 10 times by a 0.25 M sucrose solution and kept on ice or mixed with cold distilled water in a 1:1 ratio (with immediate mixing) and stored 30 min at 0 °C. The reaction was stopped by the addition of 2 mL of Na-glycine buffer (pH 10.5). Aliquots of the samples (50 μl) were then used for the determination of free β-galactosidase activity. The total activity of β-galactosidase was determined under the same conditions using Triton X-100 (the final concentration of Triton X-100 = 0.1 %). Fluorescence was quantified on a Shimadzu RF-5301 PC spectrofluorimeter with an excitation wavelength of 360 nm and an emission wavelength of 460 nm, using 4-methylumbelliferon as the standard. The results were expressed as the percent of the total activity.

### Cysteine protease activity assay

Cysteine protease cathepsin B (EC 3.4.22.1) specific activity was measured in heart and liver tissue according to Barrett and Kirschke [40] using corresponding fluorogenic substrates Arg-Arg-MCA and Arg-Phe-MCA, respectively, (Sigma, USA) (MCA = methylcoumarylamide) at pH 6.0 (0.2 M phosphate buffer) for cathepsin B as previously reported [5]. Incubation was performed in the presence of 0.1 % Triton X-100 to destroy the cellular membranes. The reaction was stopped by adding 0.1 M monochloracetic acid. Fluorescence measurements were recorded on a Shimadzu RF-5301 PC spectrofluorometer using excitation and emission wavelengths of 355 nm and 460 nm, respectively. MCA served as the standard. The results were expressed as μmol of MCA cleaved per min per g of protein. In the cathepsin L assay, a specific inhibitor of cathepsin B (CA-074, 0.3 μM) was added to the incubation medium to exclude the activity of cathepsin B.

### Morphological evaluation of liver and heart tissue

For morphological analysis of liver and heart tissue, samples were fixed with 10 % neutral-buffered formalin and prepared using a MICROM HM 340E rotational microtome (Carl Zeiss, Germany). Specimens were embedded in histoplast and 4-μm cross-sections of tissues were stained with hematoxylin and eosin according to commonly-accepted procedures. The slices were evaluated using light microscopy with an Axioscop 40 microscope (Carl Zeiss, Germany). To more completely evaluate lipid storage in liver, specimens were dehydrated and embedded in polyethylene glycol-1500 and the sections subsequently stained with Sudan III and hematoxylin.

### Statistical analysis

All values were reported as the mean ± the standard deviation (SD). Mean values of lipoprotein fractions and *sub*fractions within the various groups (LP-C, LP-TG and LP-PL) were analyzed for statistically significant differences with the software program STATISTICA 10.0 using a one-way, analysis-of-variance (ANOVA). *Post-hoc* analysis of ANOVA testing was performed using the Least Significant Difference (LSD) test. When comparing only two mean values, we used the Student’s *t*-test to identify a difference that was statistically significant. All statistical results using either ANOVA, or the Student’s *t*-test, in which *p* < 0.05 were deemed statistically significantly different and were noted in figures and tables. Correlation analysis was performed using the Spearman test [41].

## Abbreviations

ALT: alanine aminotransferase
AST: aspartate aminotransferase
HDL: high-density lipoprotein
IDL: intermediate-density lipoprotein
LDL: low-density lipoprotein
LP-C: lipoprotein cholesterol
LP-PL: lipoprotein phospholipids
LP-TG: lipoprotein triglycerides
ox-LDL: oxidized low-density lipoprotein
P-407: poloxamer 407
SAXS: small-angle X-ray scattering
TG: triglycerides
VLDL: very-low-density lipoprotein
